# Floral Volatile Compounds of *Passiflora edulis* Sims and *Poincianella pyramidalis* (Tul.) L.P. Queiroz and Their Detection by *Xylocopa* (*Neoxylocopa*) *nigrocincta* Smith (Hymenoptera: Apidae)

**DOI:** 10.3390/insects17090926

**Published:** 2026-09-04

**Authors:** Fernanda Stefanny Lima Sobrinho, Luciano Santos Medeiros, Amanda Lima Cunha, Mayara Camila Santos Silva, César Gonçalves dos Santos, Josias Jordão Andrade Alves, João Gomes da Costa, Henrique Fonseca Goulart, Antônio Euzébio Goulart Santana

**Affiliations:** 1 Laboratory of Natural Product Research, Campus for Engineering and Agrarian Sciences, Federal University of Alagoas, Maceió 57072-900, AL, Brazil; stefanny.liima@hotmail.com (F.S.L.S.);; 2 Brazilian Agricultural Research Company—Embrapa Alimentos e Territórios, Maceió 57020-050, AL, Brazil

**Keywords:** chemical ecology, cross-pollination, plant–insect communication, generalist bee

## Abstract

Floral volatile organic compounds (VOCs) play a key role in chemical communication between plants and pollinators. Given the limited understanding of the chemical signals involved in the interactions among *Passiflora edulis* Sims (Passifloraceae), *Poincianella pyramidalis* (Tul.) L.P. Queiroz (Fabaceae), and *Xylocopa (Neoxylocopa) nigrocincta* Smith (Hymenoptera: Apidae), this study aimed to identify the floral VOCs emitted by these plant species and evaluate their detection by the antennae of *X. nigrocincta*. Floral VOCs were collected using dynamic headspace sampling, adsorbed in Porapak Q, and eluted with 500 µL of hexane. The resulting extracts were analyzed by gas chromatography with flame ionization detection (GC-FID), gas chromatography–mass spectrometry (GC–MS), and gas chromatography coupled with electroantennographic detection (GC–EAD). A total of 23 compounds were identified, six of which were shared by both plant species. The chemical profiles differed markedly: *P. edulis* was characterized mainly by methoxylated ethers and esters, whereas *P. pyramidalis* showed a predominance of terpenes. 1,4-dimethoxybenzene, the major compound in *P. edulis*, and (E)-β-ocimene, the most abundant compound in *P. pyramidalis*, elicited electroantennographic responses in *X. nigrocincta*, demonstrating that both compounds are detected by the olfactory system of these bees. These findings reveal differences in the floral VOC profiles of the two plant species and identify compounds detected by *X. nigrocincta*, providing a basis for future behavioral studies to investigate the role of these volatiles in plant–pollinator interactions.

## Simple Summary

We identified a series of volatile organic compounds (VOCs) from the flowers of *Passiflora edulis* Sims (Passifloraceae) and *Poincianella pyramidalis* (Tul.) L.P. Queiroz (Fabaceae), noting marked differences in their compositions. The compounds 1,4-dimethoxybenzene and (E)-β-ocimene were the most abundant in *P. edulis* and *P. pyramidalis*, respectively. Furthermore, we investigated the responses of the generalist bee *Xylocopa* (*Neoxylocopa*) *nigrocincta* Smith (Hymenoptera: Apidae) to both compounds. The study revealed that both compounds elicit electroantennographic responses in *X. nigrocincta*, providing a basis for elucidating the behavior of this pollinator and contributing to strategies aimed at enhancing pollination in these and other species.

## 1. Introduction

Floral volatile organic compounds (VOCs) are important chemical signals mediating plant–pollinator interactions, contributing to the location, recognition, and discrimination of floral resources by insects [1,2]. The composition of these emissions may vary among plant species, resulting in specific chemical profiles that provide information to floral visitors and may influence their perception and foraging behavior [2,3]. Thus, floral chemical communication represents an important component of plant–visitor interactions and may directly influence pollination processes.

Pollination is an essential ecosystem service that supports plant reproduction, biodiversity maintenance, and agricultural production, thereby contributing to global food security [4,5]. Among pollinators, bees play a prominent role because of their frequent flower visitation and efficiency in pollen transfer [6]. The location and selection of floral resources by these insects are influenced by multiple stimuli, including floral olfactory cues, highlighting the importance of VOCs in chemical communication between plants and pollinators [3].

Among bees, species of the genus *Xylocopa* interact with a wide diversity of plants and are particularly relevant to the pollination of species whose floral traits favor large-bodied visitors for efficient pollen transfer [7,8,9]. In *Passiflora edulis* Sims (Passifloraceae), a crop of considerable economic importance, cross-pollination is critical for fruit set, and *Xylocopa* sp. bees are among its main pollinators [8,10,11]. *Poincianella pyramidalis* (Tul.) L.P. Queiroz (Fabaceae), in turn, is a tree species endemic to the Caatinga that provides floral resources to visitors and participates in plant–pollinator interactions characteristic of this ecosystem, contributing to the reproduction and maintenance of native vegetation [12,13,14,15].

Considering the importance of chemical signals in plant–pollinator interactions and the dependence of these plant species on cross-pollination, we hypothesized that *P. edulis* and *P. pyramidalis* emit VOCs that are detected by *Xylocopa* (*Neoxylocopa*) *nigrocincta* Smith (Hymenoptera: Apidae). Accordingly, the objectives of this study were to (i) characterize and compare the floral VOC profiles of *P. edulis* and *P. pyramidalis*; and (ii) identify the compounds that elicit electrophysiological responses in the antennae of *X. nigrocincta*.

## 2. Materials and Methods

### 2.1. Experimental Area

Volatile organic compound samples were collected at two distinct locations in the state of Alagoas, Brazil. The first location was an experimental *P. edulis* cultivation site situated on the A.C. Simões campus of the Federal University of Alagoas (UFAL) in Maceió, Alagoas, Brazil (9°33′13.6″ S, 35°46′15.0″ W). The region’s climate is classified as tropical rainy, featuring a dry season in the summer and a rainy period concentrated in the autumn and winter. Sampling was conducted in March 2024, when the recorded average temperature was 25.0 °C. The second location involved *P. pyramidalis* plants used for landscaping on the UFAL campus in the city of Arapiraca, located in the Agreste region of Alagoas, Brazil (9°45′58″ S, 35°38′58″ W), at an altitude of 264 m. Sampling was conducted in December 2024, when the recorded average temperature was 26.5 °C.

### 2.2. Collection of Floral Organic Compounds

**Figure 1:**
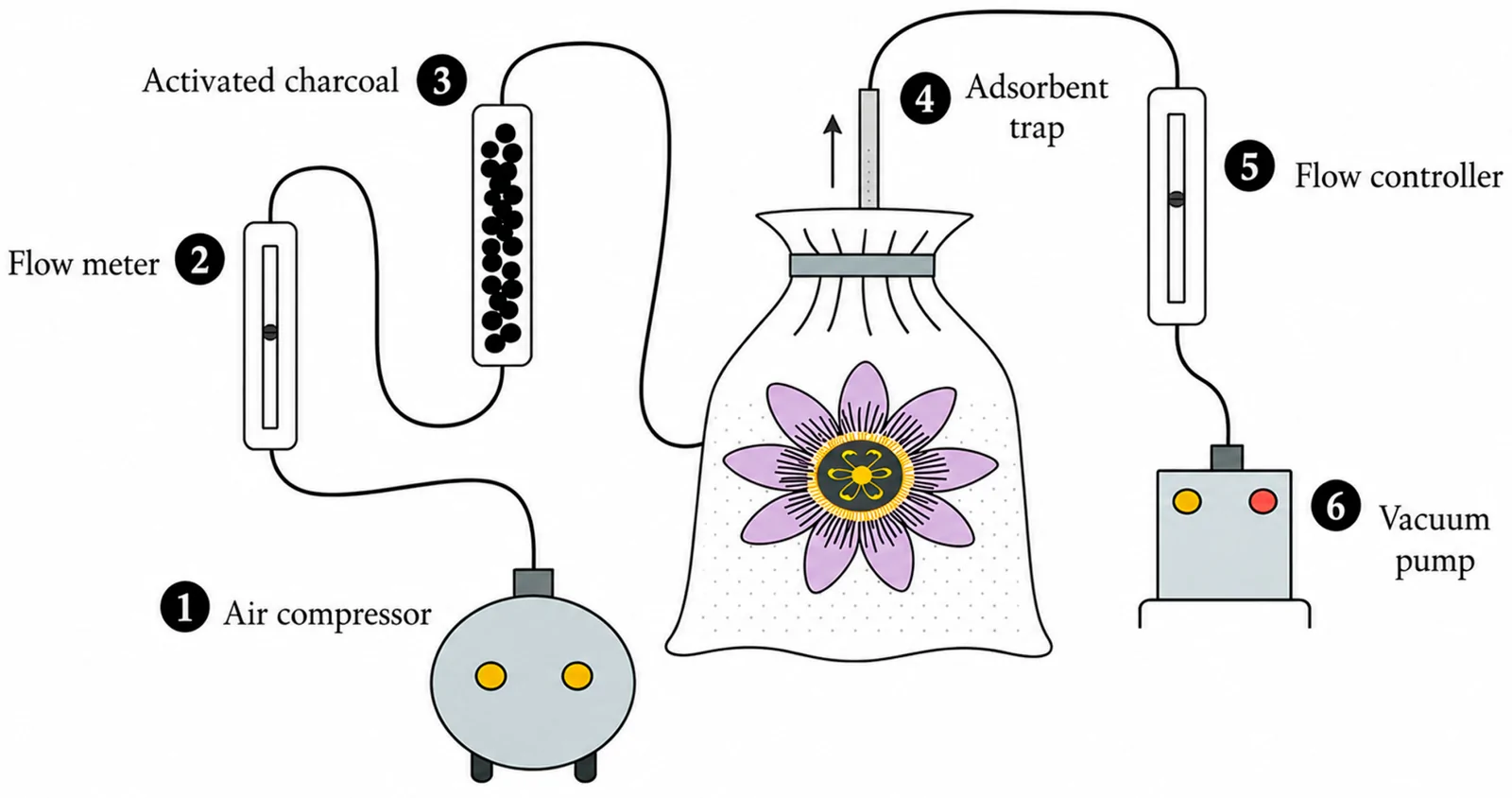
Dynamic headspace system for collecting volatile compounds from a flower. The arrow indicates the airflow direction toward the adsorbent tube.

VOCs emitted from *P. edulis* and *P. pyramidalis* flowers were collected using dynamic headspace sampling [16]. Individual flowers were enclosed in polyester bags (33 cm × 41 cm), into which air supplied by an Air Pump 660 (Jebo, Monterey Park, CA, USA) was introduced after passing through an activated charcoal filter (Merck KGaA, Darmstadt, Germany) at a flow rate of 400 mL/min. At the outlet of the system, the air carrying the floral VOCs was drawn through tubes containing Porapak Q (80/100 mesh, 0.05 g; Supelco, Bellefonte, PA, USA), which served as the adsorbent. The outlet flow rate was maintained at 350 mL/min using a flow controller connected to a vacuum pump, thereby ensuring a continuous airflow through the collection system (Figure 1).

Collections were conducted over a 4 h period, starting at 1:00 p.m. for *P. edulis* and at 8:00 a.m. for *P. pyramidalis*. Collection times were selected based on prior field observations of *X. nigrocincta* visitation periods to the flowers of each plant species. Before sampling, the Porapak Q tubes were conditioned with hexane and heated at 100 °C for 120 min in adapted ovens to activate the adsorbent. After each 4-h collection period, VOCs adsorbed onto the Porapak Q were eluted with 500 µL of HPLC-grade hexane (Sigma-Aldrich, St. Louis, MO, USA). The resulting extracts were transferred to glass vials and stored at −20 °C until chromatographic analysis.

### 2.3. Analysis of Volatile Organic Compounds

#### 2.3.1. Gas Chromatography Coupled with Flame Ionization Detection (GC-FID)

Chromatographic analysis of the VOCs was performed at the Natural Resources Research Laboratory (LPqRN) of the Federal University of Alagoas, A.C. Simões campus. For gas chromatography analysis, 1 μL of the plant extracts was injected. The analysis was conducted using a Shimadzu GC-2010 PLUS gas chromatograph (Shimadzu Corporation, Kyoto, Japan) equipped with a flame ionization detector (FID). The capillary column used was an NST-615 (30 m × 0.25 mm × 0.25 μm; Restek Chromatography Products, Bellefonte, PA, USA). A splitless injection mode was used, with the injector temperature set at 250 °C. The initial oven temperature was 50 °C, held for 5 min. Subsequently, the temperature was raised to 250 °C at a rate of 8 °C/min and then to 300 °C at a rate of 10 °C/min, holding at 300 °C for 10 min. The carrier gas used was nitrogen (N_2_), with a flow rate of 1.21 mL/min through the column. Sample injection was performed manually. The solvent used for sample preparation was HPLC-grade hexane (Merck KGaA, Darmstadt, Germany).

#### 2.3.2. Gas Chromatography–Mass Spectrometry (GC-MS)

Aliquots (1 µL) of each extract obtained from *P. edulis* and *P. pyramidalis* flowers were used. The analysis was performed on a Shimadzu 2010 Plus gas chromatograph coupled to a Shimadzu QP-2010 mass spectrometer (Shimadzu Corporation, Kyoto, Japan), equipped with an NST-01 column (30 m × 0.25 mm i.d. × 0.25 μm). Ionization was achieved via electron impact at 70 eV. The GC-MS operated in splitless mode; the injector temperature was 250 °C, and the initial oven temperature was 50 °C, held for 5 min. Subsequently, the temperature was raised to 220 °C at a rate of 6 °C/min, and then to 280 °C at a rate of 10 °C/min. Helium was used as the carrier gas, with a column flow rate of 1.10 mL/min.

### 2.4. Compound Identification

Volatile compounds were identified through spectral analysis and the use of Kovats Indices (KI), calculated for each component using a homologous series of n-alkanes (C7–C30) (Sigma-Aldrich, St. Louis, MO, USA). The obtained values were compared with literature references to assist in compound characterization. Additionally, the mass spectra of the extract components were analyzed and compared with spectral data from the National Institute of Standards and Technology (NIST) libraries (NIST08 and NIST08s) [17] and the Wiley MS library. To ensure accuracy in the identification of volatile compounds, the obtained data were compared with other literature sources, including the Pherobase [18] and NIST Chemistry WebBook [19] websites. Whenever possible, the identity of the compounds was confirmed by comparison with commercial standards or standards synthesized in the laboratory.

### 2.5. Gas Chromatography Coupled with Electroantennography (GC-EAD)

Gas chromatography coupled with electroantennography (GC-EAD) was used to identify electrophysiologically active compounds in the extracts. Aliquots of 2–4 μL of the extracts obtained from *P. edulis* and *P. pyramidalis* were injected into a gas chromatograph coupled to a flame ionization detector (FID), model QP-2010 (Shimadzu Corporation). Analyses were performed in splitless mode using an RTX-5 capillary column (30 m × 0.25 mm × 0.25 μm) (Restek Chromatography Products, Bellefonte, PA, USA). The column effluent was split into two outlets: one directed to the FID for recording chromatographic signals, and the other led to the electroantennography (EAD) system, allowing for simultaneous monitoring of antennal responses to the eluted compounds. The injector temperature was maintained at 250 °C. The oven temperature program began at 50 °C (held for 5 min), followed by heating to 90 °C at a rate of 15 °C/min, then to 160 °C at 6 °C/min, and finally to 280 °C at 20 °C/min (held for 5 min). The carrier gas flow rate was maintained at 1.90 mL/min. The antennae of female *X. nigrocincta* were removed at the base of the scape after cold anesthesia in a freezer for approximately 5 min. Signals were transmitted via a high-impedance amplifier (IDAC4, Syntech 2004, Hilversum, The Netherlands). The outputs from the EAD amplifier and the GC-FID were monitored simultaneously and analyzed using the GcEad32 software package, version 4.6. Peaks resulting from the GC run were considered active when they elicited EAD activity in at least three replicates.

## 3. Results

**Figure 2:**
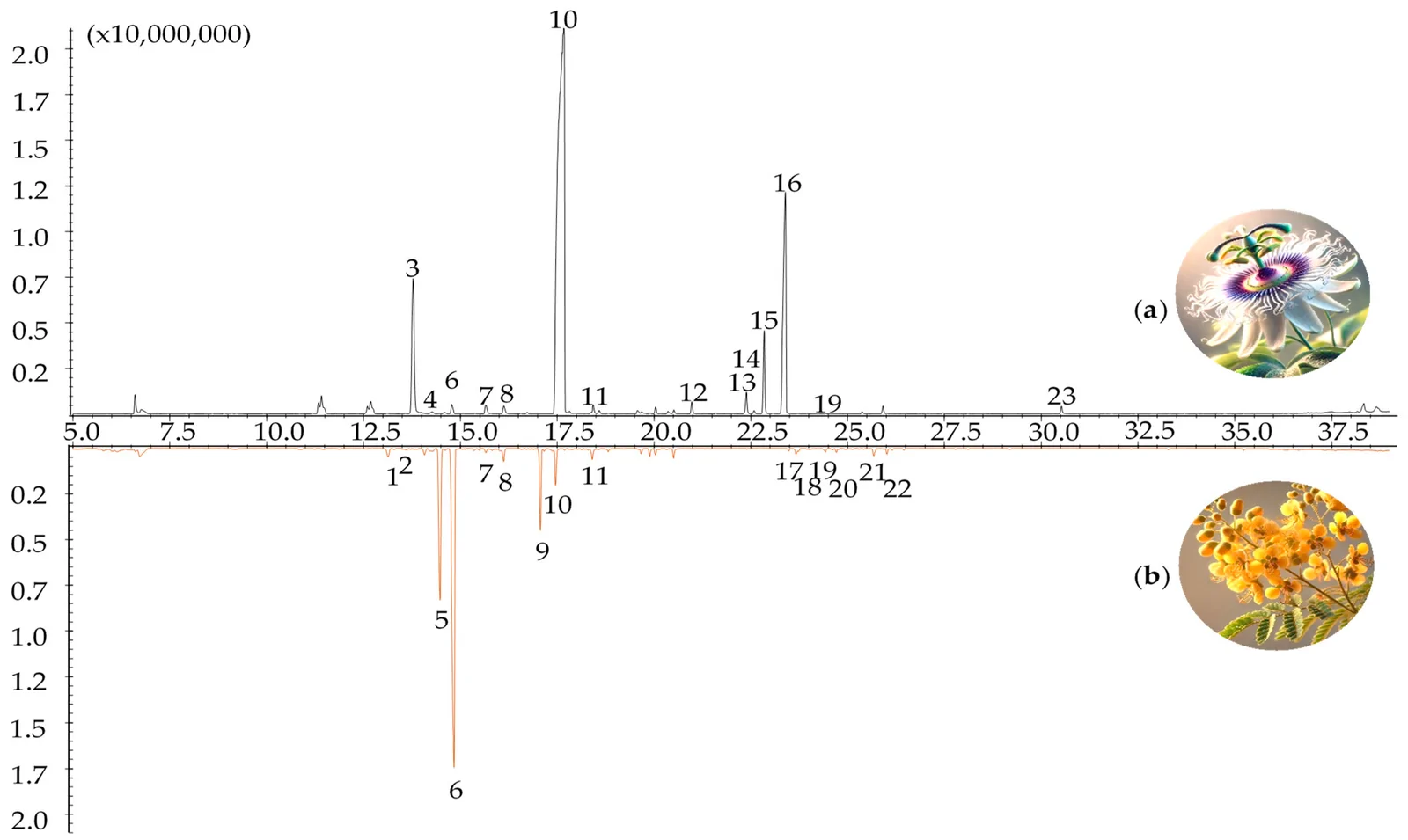
Chemical composition of the flowers of (**a**) *Passiflora edulis* and (**b**) *Poincianella pyramidalis*.

Analysis of the extracts revealed the presence of 23 compounds: 14 in the *P. edulis* flower extract and 15 in the *P. pyramidalis* flower extract. Of these, six were common to both species: (*E*)-β-ocimene (**6**), methyl benzoate (**7**), linalool (**8**), 1,4-dimethoxybenzene (**10**), methyl salicylate (**11**), and caryophyllene (**19**) (Figure 2).

Table 1 lists the compounds identified in the volatile extracts obtained from *P. edulis* and *P. pyramidalis* flowers. Terpenes predominate in the volatile composition of *P. pyramidalis*, whereas passion fruit flowers showed a higher abundance of aromatic ethers. Among the detected compounds, alcohols and esters were identified in both species. In *P. edulis* flowers, the major compound was 1,4-dimethoxybenzene (**10**), followed by 1,3,5-trimethoxybenzene (**16**) and benzyl alcohol (**3**). Esters were also present, notably methyl (Z)-3-phenyl-2-propenoate (**15**), methyl cinnamate (**12**), methyl benzoate (**7**), and methyl salicylate (**11**). Regarding terpenes, (*E*)-β-ocimene (**6**), linalool (**8**), limonene (**4**), and caryophyllene (**19**) were identified. *P. pyramidalis* flowers exhibited a volatile chemical composition characterized by terpenes, with (*E*)-β-ocimene (**6**) and *cis*-ocimene (**5**) standing out as the major compounds. Other identified terpenes include allo-ocimene (**9**), β-myrcene (**1**), linalool (**8**), and caryophyllene (**19**), as well as copaene (**17**), β-bourbonene (**18**), α-bergamotene (**20**), D-germacrene (**21**), and farnesene (**22**) in smaller proportions. The identified esters were methyl benzoate (**7**) and methyl salicylate (**11**), while 1,4-dimethoxybenzene (**10**) was detected at low concentration.

**Figure 3:**
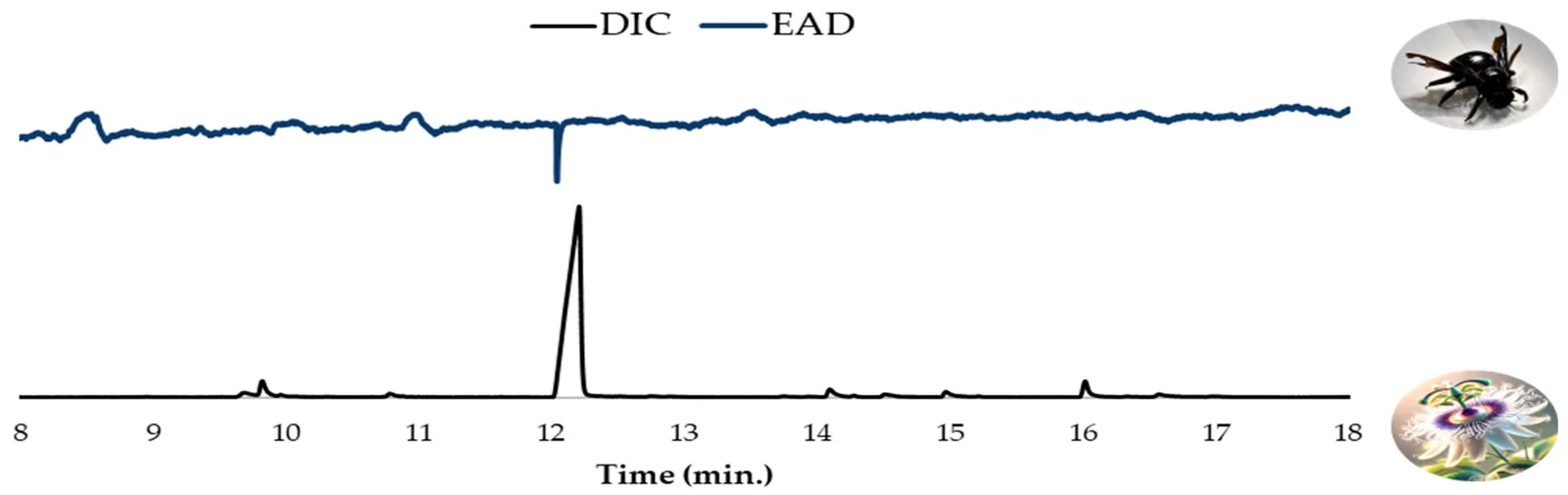
Electroantennographic response of the bee *X. nigrocincta* to 1,4-dimethoxybenzene, the main volatile compound detected in *Passiflora edulis* flowers.

The electroantennography test revealed specific electrophysiological responses of female *X. nigrocincta* to certain compounds present in the flower extracts of *P. edulis* and *P. pyramidalis*. A pronounced electroactive response was observed to 1,4-dimethoxybenzene, identified as the major constituent of the volatile extract from passion fruit flowers (Figure 3).

**Figure 4:**
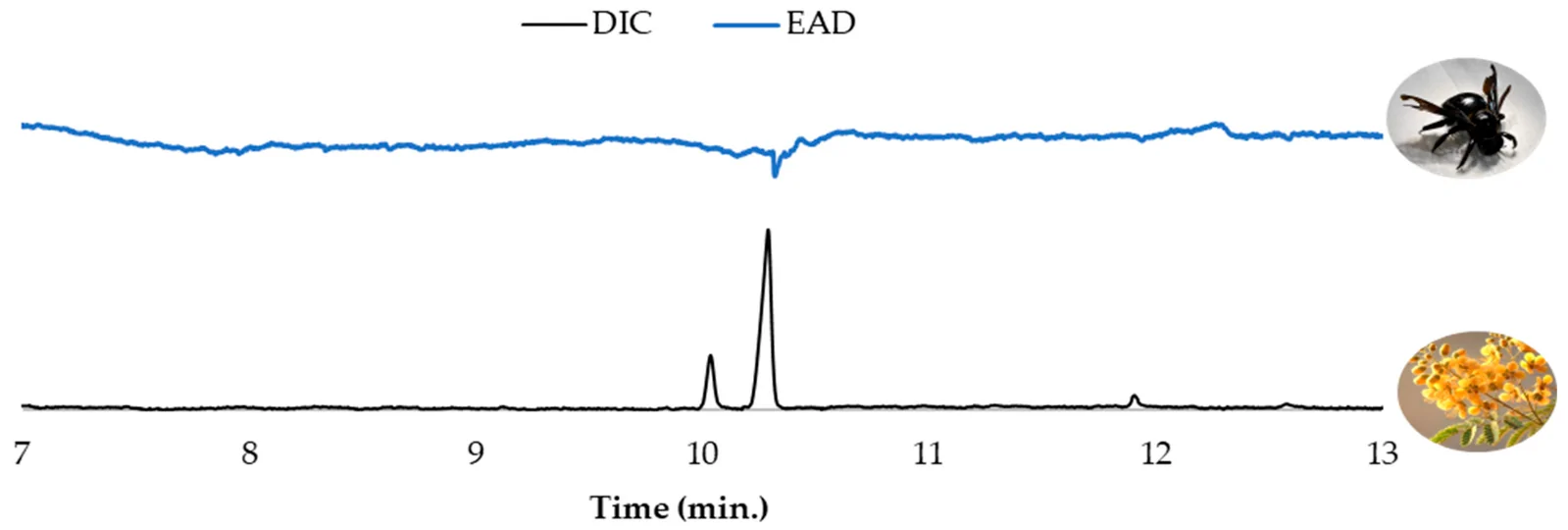
Electroantennographic response of *X. nigrocincta* to (*E*)-β-ocimene, the major constituent of the floral volatiles of *Poincianella pyramidalis*.

Furthermore, electroantennographic activity was assessed for (*E*)-β-ocimene, the major floral compound of *P. pyramidalis* (Figure 4).

## 4. Discussion

The volatile organic compound composition revealed that *P. edulis* and *P. pyramidalis* exhibit distinct floral chemical profiles, although they share six compounds: (*E*)-β-ocimene, methyl benzoate, linalool, 1,4-dimethoxybenzene, methyl salicylate, and β-caryophyllene. These volatiles are among the most frequently occurring compounds in angiosperm flowers and play important roles in chemical communication between plants and pollinators [20]. However, the main difference between the two species lies primarily in the relative abundance of these constituents. In general, floral chemical identity is determined by the combination and relative proportions of the emitted compounds, which can influence recognition by floral visitors [21,22].

Flowers emit complex blends of volatile organic compounds that may play different roles in plant–pollinator interactions. Although many of these compounds are shared among plant species, some predominant constituents may act as chemical signals more characteristic of individual species. In this context, *P. edulis* and *P. pyramidalis* exhibited distinct profiles, with 1,4-dimethoxybenzene predominating in *P. edulis* and (*E*)-β-ocimene in *P. pyramidalis*. In addition to these predominant compounds, both species shared linalool, methyl benzoate, methyl salicylate, and β-caryophyllene, compounds that are widely distributed in floral scents. Linalool is one of the most common monoterpenes in bee-visited flowers and has been associated with the attraction of diverse groups of pollinators [21]. The benzenoids methyl benzoate and methyl salicylate are also commonly found in entomophilous species and contribute to chemical communication between plants and insects [20,23], whereas β-caryophyllene has been associated with the attraction of different floral visitors, including bees [24].

In *P. edulis*, 1,4-dimethoxybenzene was the predominant compound, consistent with the chemical profile previously reported [25], which identified it as the major constituent of the floral scent of this species. Other methoxylated derivatives, benzyl alcohol, and several aromatic esters were also detected, resulting in a profile dominated by oxygenated aromatic compounds. Although these compounds are frequently found in floral scents [20,26], the present study provides additional functional evidence by demonstrating that 1,4-dimethoxybenzene is detected by the antennae of *X. nigrocincta*. This compound has previously been reported as attractive to different bee species, including *Andrena vaga* Panzer, 1799 (Hymenoptera: Adrenidae) [27] and Euglossini species [28], further supporting its ecological relevance in mediating interactions between flowers and pollinators.

In contrast, *P. pyramidalis* exhibited a chemical profile characterized primarily by the emission of (*E*)-β-ocimene and (*Z*)-β-ocimene, together with allo-ocimene and other mono- and sesquiterpenes. β-ocimene is considered one of the most widespread floral volatile compounds, occurring across a large proportion of the plant families studied [20], and has also been implicated in the attraction of bees, butterflies, moths, and other floral visitors [29]. The high relative abundance of (*E*)-β-ocimene in *P. pyramidalis*, together with the antennal response observed in this study, supports its potential role as one of the major chemical signals involved in floral recognition by *X. nigrocincta*. Furthermore, the predominance of terpenes observed in this species is consistent with the role of these metabolites in mediating ecological interactions between plants and insects [30,31].

Although an electroantennographic response alone does not demonstrate behavioral attraction, it confirms the sensory detection of these compounds and represents an essential step toward identifying chemical signals potentially involved in plant–pollinator interactions [21,22].

## 5. Conclusions

The results revealed distinct chemical signatures: *P. edulis* flowers were characterized by a predominance of aromatic ethers, with 1,4-dimethoxybenzene as the major constituent, whereas *P. pyramidalis* exhibited a predominantly terpenoid profile, with (*E*)-β-ocimene as the major compound. Electroantennographic analyses demonstrated that *X. nigrocincta* detects both compounds, providing evidence of their antennal perception. Although the two plant species share some constituents, each exhibits a characteristic volatile profile, reinforcing the importance of VOCs in chemical communication between plants and pollinators. Future studies involving behavioral bioassays and field experiments may clarify the roles of 1,4-dimethoxybenzene and (***E***)-β-ocimene in interactions with *X. nigrocincta,* as well as assess their potential for the development of strategies aimed at pollinator attraction and pollination management in production systems.

## Figures and Tables

**Table 1:** Volatile compounds detected in the flowers of *Poincianella pyramidalis* and *Passiflora edulis*.

No.	RT (min)	LRI	Compound	Common Name	Functional Group	*P. pyramidalis* (%)	*P. edulis* (%)
1	13.135	983	7-methyl-3-methylene-1,6-octadiene *	β-myrcene	Hydrocarbon (Monoterpene)	1.31	-
2	13.289	988	(*E*)-3-hexen-1-yl acetate	cis-3-hexenyl acetate	Ester	0.10	-
3	13.777	1004	Phenylmethanol *	Benzyl alcohol	Alcohol	-	8.85
4	14.268	1021	1-methyl-4-(prop-1-en-2-yl)cyclohexene *	Limonene	Hydrocarbon (Monoterpene)	-	0.12
5	14.476	1028	(*Z*)-3,7-dimethyl-1,3,6-octatriene *	(*Z*)-β-ocimene	Hydrocarbon (Monoterpene)	22.73	-
6	14.838	1041	(*E*)-3,7-dimethyl-1,3,6-octatriene *	(*E*)-β-ocimene	Hydrocarbon (Monoterpene)	57.27	0.47
7	15.659	1069	Methyl benzoate *	Methyl benzoate	Ester	0.45	0.49
8	16.117	1085	3,7-dimethyl-1,6-octadien-3-ol *	Linalool	Monoterpene alcohol	1.54	0.45
9	17.069	1119	2,6-dimethyl-2,4,6-octatriene *	allo-ocimene	Hydrocarbon (Monoterpene)	8.57	-
10	17.490	1135	1,4-dimethoxybenzene *	1,4-dimethoxybenzene	Ether	3.76	68.51
11	18.411	1170	Methyl 2-hydroxybenzoate *	Methyl salicylate	Ester	1.25	0.33
12	20.973	1272	Methyl (2*E*)-3-phenylprop-2-enoate	Methyl cinnamate	Ester	-	0.50
13	22.387	1332	1,2,4-trimethoxybenzene	1,2,4-trimethoxybenzene	Ether	-	0.91
14	22.588	1340	Methyl 4-methoxybenzoate	Anisic acid methyl ester	Ester	-	0.11
15	22.844	1352	Methyl (2*Z*)-3-phenylprop-2-enoate	(*Z*)-methyl cinnamate	Ester	-	3.46
16	23.393	1376	1,3,5-trimethoxybenzene *	1,3,5-trimethoxybenzene	Ether	-	15.46
17	23.480	1380	(1*R*,2*S*,6*S*,7*S*,8*S*)-8-Isopropyl-1,3-dimethyltricyclo[4.4.0.0^2^,^7^]dec-3-ene	Copaene	Hydrocarbon (Sesquiterpene)	0.24	-
18	23.670	1388	1-methyl-5-methylidene-8-(propan-2-yl)tricyclo[5.3.0.0^2^,^6^]decane	β-bourbonene	Hydrocarbon (Sesquiterpene)	0.67	-
19	24.427	1422	4,11,11-trimethyl-8-methylenebicyclo[7.2.0]undec-4-ene *	β-caryophyllene	Hydrocarbon (Sesquiterpene)	0.41	0.02
20	24.708	1436	2,6-dimethyl-6-(4-methylpent-3-enyl)bicyclo[3.1.1]hept-2-ene	α-bergamotene	Hydrocarbon (Sesquiterpene)	0.29	-
21	25.676	1481	1,5-dimethyl-8-(prop-1-en-2-yl)cyclodeca-1,5-diene	Germacrene D	Hydrocarbon (Sesquiterpene)	0.93	-
22	26.014	1497	3,7,11-trimethyldodeca-1,3,6,10-tetraene *	Farnesene	Hydrocarbon (Sesquiterpene)	0.48	-
23	30.528	1728	Benzyl benzoate *	Benzyl benzoate	Ester	-	0.32

RT = retention time; LRI = calculated linear retention index; (*) = compound confirmed by comparison with an authentic synthetic standard.

## Data Availability

The data presented in this study are available in the article.
